# SARS-CoV-2 innate immune recognition and implications for respiratory health

**DOI:** 10.1016/j.cytogfr.2025.10.008

**Published:** 2025-10-28

**Authors:** Vandana Anang, Pankaj Kumar, Jamaluddin Pracha, Richard S. Nho, Ana L. Mora, Mauricio Rojas, Kymberly Gowdy, Jacob S. Yount, Joseph S. Bednash, Jeffrey C. Horowitz, Sourabh Soni, Yohannes A. Mebratu

**Affiliations:** a Department of Internal Medicine, Division of Pulmonary, Critical Care and Sleep Medicine, Davis Heart and Lung Institute, College of Medicine, The Ohio State University Wexner Medical Center, Columbus, OH, USA; b Department of Microbial Infection and Immunity, College of Medicine, The Ohio State University Wexner Medical Center, Columbus, OH, USA

**Keywords:** SARS-CoV-2, Innate Immunity, Pattern Recognition Receptors (PRRs), Interferon (IFN) Response, Cytokine Storm, COVID-19 Pathogenesis, Respiratory Health

## Abstract

The ongoing global health impact of SARS-CoV-2, particularly on lung and respiratory health, underscores the critical need to decipher the intricate interplay between the virus and the host innate immune system. This review provides an analysis of the key pattern recognition receptors (PRRs) involved in SARS-CoV-2 recognition within the lung, including toll-like receptors (TLRs), RIG-I-like receptors (RLRs), NOD-like receptors (NLRs), and C-type lectin receptors (CLRs). We discuss how the engagement of these innate sentinels triggers crucial downstream consequences, ranging from protective antiviral interferon (IFN) responses to detrimental hyperinflammation characteristic of severe COVID-19. Numerous studies have identified sophisticated mechanisms employed by SARS-CoV-2 to evade or suppress early IFN induction, contributing to unchecked viral replication and subsequent immunopathology. We explore how this aberrant innate immune response drives the "cytokine storm", leading to acute respiratory distress syndrome (ARDS) and long-term sequelae. Furthermore, this review critically assesses current and emerging therapeutic strategies aimed at modulating innate immunity, including TLR agonists/antagonists, RIG-I/MDA5 modulators, NLRP3 inflammasome inhibitors, and IFN-based therapies, highlighting their potential and associated challenges. Finally, we identify key research gaps, emphasizing the need for cell-type-specific PRR studies, comprehensive mapping of viral evasion mechanisms, and the development of precision immunotherapies to enhance protective responses and mitigate pathogenic inflammation for future respiratory viral threats.

## 1. Introduction

The severe acute respiratory syndrome coronavirus 2 (SARS-CoV-2), the causative agent of COVID-19, continues to exert a significant global health impact, particularly on lung and respiratory health. Years after the acute COVID-19 pandemic caused by SARS-CoV-2, the virus continues to exert a significant global impact, particularly on lung and respiratory health [1,2]. While the pandemic emergency was officially lifted in May 2023, the virus remains active in global circulation [3].

Sentinel surveillance data from February 2025 indicates a renewed increase in SARS-CoV-2 activity, with global test positivity reaching 11 %, a peak not seen since July 2024. This resurgence is linked to the constant emergence of new variants, such as the Variant Under Monitoring (VUM) NB.1.8.1, which underscores the virus’s persistent ability to evolve and evade immunity [3].

A major challenge in control is the virus’s unpredictable nature; unlike influenza, SARS-CoV-2 lacks clear seasonality, complicating public health planning. Furthermore, reduced global genomic surveillance hinders real-time understanding of variant spread [4]. The enduring impact on health is also seen in the long-term consequences of SARS-CoV-2 infection, including post-acute sequelae (long COVID), persistent inflammation, and immune dysregulation [5].

Biologically, SARS-CoV-2 is highly adept at manipulating host systems. It enters human cells primarily *via* the angiotensin-converting enzyme 2 (ACE2) receptor, though several other host proteins, including neuropilin-1, C-type lectins, basigin (CD147), and tyrosine kinase receptors, can facilitate viral entry [6]. To establish infection and evade immune responses, the virus disrupts key antiviral pathways, particularly the type I interferon (IFN) response [7].

The innate immune system is the body’s rapid, first line of defense, activating within minutes to hours to limit viral spread, particularly in the upper respiratory tract [8]. The respiratory epithelium, acting as both a physical barrier and an active immune interface, plays a pivotal role in this defense. It promptly detects viral components through pattern recognition receptors (PRRs), including toll-like receptors (TLRs) such as TLR3 and TLR7/8 that recognize viral RNA, and RIG-I-like receptors (RIG-I and MDA5) [9].

Several key innate immune cells play significant roles in viral control within the lungs. Alveolar macrophages (AMs) rapidly phagocytose viruses and infected cells, thereby limiting the initial infection and spread [10]. Dendritic cells (DCs) capture and present viral antigens to T and B cells, effectively bridging innate and adaptive immune responses. Concurrently, Natural Killer (NK) cells directly target and eliminate infected host cells without requiring prior sensitization [11].

To develop effective countermeasures, it’s vital to understand these initial host-virus interactions. This review provides an in-depth overview of the key PRRs involved in SARS-CoV-2 recognition, focusing on the critical context of lung and respiratory health. We discuss the implications of innate immune recognition for viral pathogenesis and explore potential therapeutic strategies that modulate these fundamental immune pathways.

## 2. Key pattern recognition receptors (PRRs) involved in SARS-CoV-2 detection

**Fig. 1.**
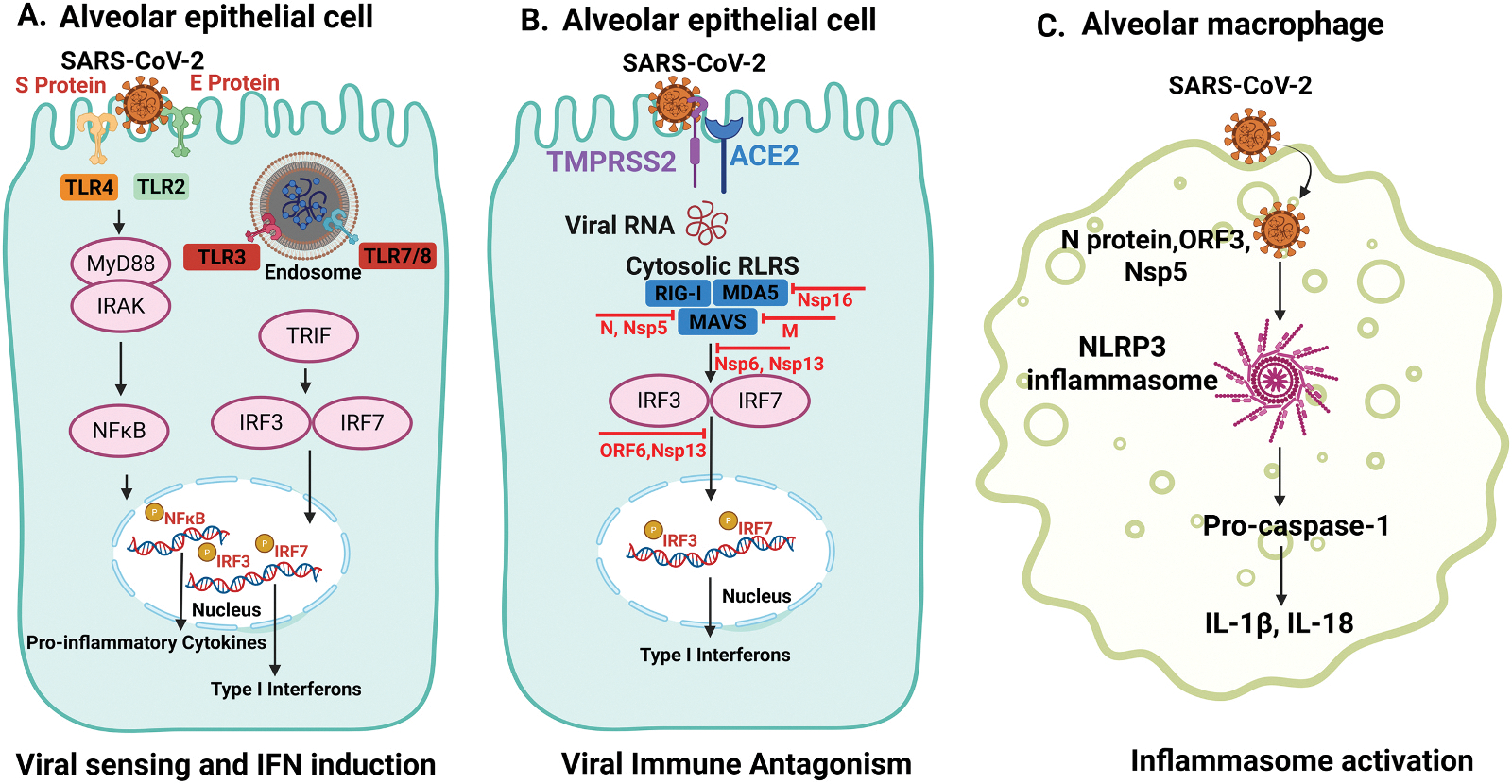
Dynamic interplay between SARS-CoV-2 and the host innate immune system in the lung. (A). Spike (S) and Envelope (E) proteins of SARS-CoV-2 are detected by surface Toll-like receptors (TLRs), TLR4 and TLR2; endosomal TLRs, TLR3, TLR7, and TLR8 on alveolar epithelial cells (AECs). These sensors activate MyD88 and TIR domain containing adaptor molecule 1 (TRIF), respectively, leading to NF-κB and IRF3/7 activation, resulting in the production and pro-inflammatory cytokines and type I interferons (IFN-I). (B) The virus enters the AECs *via* ACE2 and TMPRSS2. Viral RNA intermediates are recognized by cytosolic RIG-I-like receptors (RLRs), RIG-I and MDA5. These sensors activate IRF3/7 and lead to the production of IFN-I. SARS-CoV-2 employs multiple strategies to evade and suppress host antiviral defenses: N protein and Nsp5 cleave RLR adaptor components such as mitochondria anti-viral signaling (MAVS), and Nsp16 is involved in modifying viral RNA to evade recognition by RIG-I and MDA-5. M protein interaction with MAVS disrupts downstream signaling. Nsp6 and Nsp13 interact with the MAVS-IRF3 signaling cascade, thereby limiting IRF3 activation. ORF6 and Nsp13 impede the nuclear translocation of IRF3. (C) In alveolar macrophages, N protein, ORF3, and Nsp5 activate the NLRP3 inflammasome, promoting maturation and secretion of IL-1β and IL-18.

The innate immune system uses germline-encoded PRRs to detect conserved PAMPs and damage-associated molecular patterns (DAMPs) from host cells [12,13], triggering signaling pathways that produce pro-inflammatory cytokines [14]. Based on their localization, they are categorized as membrane-bound PRRs (e.g., TLRs and C-type lectin receptors (CLRs)) or cytoplasmic PRRs (e.g., NLRs and RLRs) [15]. Notably, TLR3 and TLR7 as well as retinoic acid-inducible gene I-like receptors (RLRs), including MDA5 and RIG-I, have been shown to contribute to the IFN response induced by coronaviruses, though this function may vary depending on the cell type involved [16]. Fig. 1 illustrates innate immune signaling pathways implicated in the response to SARS-CoV-2 infection and Table 1 provides an overview of the key PRRs in SARS-CoV-2 recognition.

### 2.1. Toll-like receptors (TLRs)

TLRs are membrane-bound PRRs that recognize microbial components like lipoproteins, lipids, and nucleic acids. They activate immune cells and induce cytokine production for pathogen clearance and for programming of proper adaptive immune responses. Given their role in modulating innate and adaptive immune response, TLRs are also explored as therapeutic targets in infectious diseases, autoimmune disorders, inflammatory conditions and cancer [12].

#### 2.1.1. TLR2

TLR2 is a membrane-bound PRR known for detecting bacterial components [17]. It also senses SARS-CoV-2 Spike (S) and Envelope (E) proteins [18,19] activating pro-inflammatory pathways that amplify the innate immune response [20]. On activation, TLR2 recruits the adaptor protein MyD88, leading to downstream activation of transcription factor NF-κB to induce the production of pro-inflammatory cytokines and chemokines (Fig. 1).

#### 2.1.2. TLR3

TLR3 recognizes viral double-stranded RNA (dsRNA), a replication intermediate of many RNA viruses, including coronaviruses. On recognition of viral dsRNA, TLR3 signals through the adaptor molecule TRIF (TIR-domain-containing adapter-inducing IFN-β), activating IRF3 and NF-κB, which in turn induce type I IFNs (primarily IFN-β) and pro-inflammatory cytokine production [21–23] (Fig. 1). In airway epithelial cells (AECs), its activation promotes early IFN-β responses, but excessive or prolonged activation in AMs and other immune cells may contribute to tissue injury and exacerbated inflammation, as observed in some cases of acute lung injury (ALI) and severe viral pneumonia [24,25].

#### 2.1.3. TLR4

TLR4, a critical receptor for bacterial lipopolysaccharide (LPS), has been implicated in the hyperinflammatory state of severe COVID-19. Initial studies suggested a direct interaction between the SARS-CoV-2 Spike (S) protein and TLR4, proposing that this binding initiated a pro-inflammatory signal in the lung endothelium and immune cells [26–28]. This leads to the upregulation of pro-inflammatory cytokines such as Interleukin-6 (IL-6), TNF-α, and IL-1β and contributing to the cytokine storm, a hyperinflammatory state characterized by the uncontrolled systemic release of cytokines [29] and pulmonary pathology seen in critical COVID-19 patients [28,30].

**Fig. 2.**
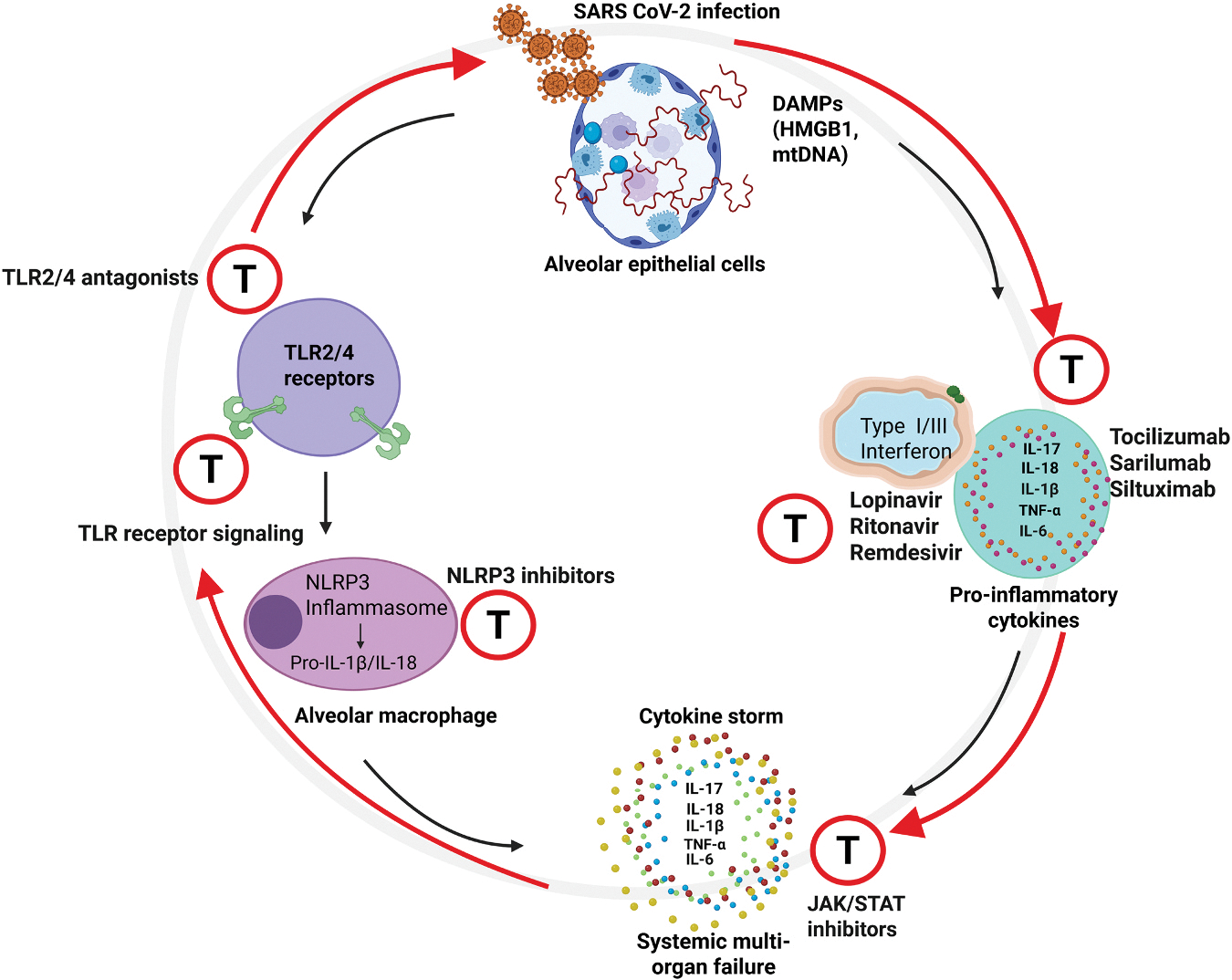
Therapeutic targeting of innate immune and inflammatory pathways during SARS-CoV-2 infection. Alveolar epithelial cells release DAMPs (Damage-associated molecular patterns) such as High Mobility Group Box 1 (HMGB1) and mitochondrial DNA (mtDNA). These DAMPs create a self-perpetuating pathogenic loop by engaging TLR2/4 (on the endothelium) and the NLRP3 inflammasome (in alveolar macrophages), leading to massive and uncontrolled production of pro-inflammatory cytokines. Additionally, DAMPs-driven activation massively amplifies the release of a diverse array of pro-inflammatory cytokines (IL-17A, IL-18, IL-1β, TNF-α, IL-6) and type I/III interferons. This leads to a cytokine storm, which drives systemic inflammation and multi-organ failure. The figure also highlights key therapeutic intervention points (marked by T in red circle) targeting pro-inflammatory cytokines, the JAK/STAT signaling pathway, DAMP sensors (TLR2/4 and NLRP3), TLR receptor inhibitors, and type I/III IFNs, to break this hyper-inflammatory feedback loop.

However, direct interaction between the S protein and TLR4 remains a subject of significant controversy, and its role in inducing TLR4-mediated inflammation requires further investigation [31,32]. These contradictory reports, often employing more biologically relevant, full-length, and properly glycosylated Spike proteins, have failed to demonstrate a direct and specific binding to human TLR4 or its accessory proteins (e.g., CD14 and MD-2) [32]. Some studies have reported conflicting findings, with recombinant S proteins purified from HEK293 cells failing to activate TLR4 in similar experimental systems [18]. A key technical caveat involves endotoxin contamination in recombinant S protein preparations (produced in both *E. coli* and human cells, CHO and HEK293), which has been suggested to be responsible for activating human macrophages. This study indicates that the S protein and ultra-low levels of LPS may act synergistically to induce macrophage activation, rather than the S protein being a direct, solitary ligand [33]. The envelope (E) protein, rather than the S protein, was found to interact with TLR4 and TLR2, triggering airway inflammation and weakening the epithelial barrier in airway epithelial cells [34]. Therefore, the current consensus leans toward an indirect mechanism for TLR4 activation in severe COVID-19. It is more likely that the pathological TLR4-mediated inflammation is driven by endogenous host factors. Specifically, DAMPs-such as high mobility group box 1 (HMGB1), mitochondrial DNA, heat shock proteins, and fibrinogen breakdown products, released from dying lung epithelial and endothelial cells serve as potent, authentic ligands for TLR4. This host-mediated TLR4 activation by DAMPs, rather than a direct viral mechanism, is a primary driver of the sustained inflammation and microvascular injury observed in the lungs of patients with severe respiratory distress [35–37] (Fig. 2).

#### 2.1.4. TLR7/8

TLR7 and TLR8 are endosomal PRRs that detect viral single-stranded RNA (ssRNA), including SARS-CoV-2 genomes. Found mainly in innate immune cells [38–40], they trigger MyD88-dependent signaling, activating NF-κB and IRF7, leading to type I IFNs and pro-inflammatory cytokine production [41–43] (Fig. 1). Importantly, loss-of-function mutations in TLR7 have been linked to severe COVID-19 in young males, highlighting its critical role in antiviral responses [44]. These findings were further supported by independent studies reporting defective type I IFN responses in patients with inborn errors of TLR7 signaling or autoantibodies against IFN-α/β [45,46]. TLR7/8 responses are a double-edged sword. While they’re crucial for promoting viral clearance through robust Type I IFN production, their excessive or dysregulated activation can lead to severe hyperinflammation and cytokine storm in COVID-19 [38,41,47]. This need to manage over-activation makes TLR7/8 a key therapeutic target for modulating inflammation [48].

The clinical significance of TLR regulation in the lungs is further underscored by SARS-CoV-2’s capacity for antagonism. Viral non-structural protein 1 (Nsp1) has been shown to block the phosphorylation of IRF3, IRF7, STAT1, and cJun, thereby directly suppressing the TLR-mediated induction of IFN and NF-κB promoters. This critical time window of delayed signaling contributes to uncontrolled viral replication and the subsequent transition to hyperinflammation. Therefore, TLRs represent a dual-function therapeutic target: while agonists may boost protective early IFN, antagonists of TLR2 and TLR4 are being actively explored to dampen the excessive, pathogenic inflammation driven by the Spike protein and DAMPs in severe pulmonary disease.

### 2.2. Cytosolic RNA sensors

Cytosolic RNA sensors are components of the innate immune system that detect viral RNA within the host cells and trigger antiviral responses. The main sensors RIG-I, melanoma differentiation-associated gene 5 (MDA5) and LGP2 recognize different RNA structures and signal through mitochondrial antiviral signaling protein (MAVS), to activate type I/III IFN and pro-inflammatory cytokine production (Fig. 1). This pathway provides rapid defense against RNA viruses, including influenza virus and SARS-CoV-2, while their dysregulation can drive excessive inflammation [49].

#### 2.2.1. RIG-I (Retinoic acid-inducible gene I)

RLRs (RIG-I-like receptors) are critical RNA sensors in innate antiviral immunity. RIG-I and MDA5 are equipped with N-terminal caspase recruitment domains and are activated by viral RNAs in the cytoplasm of infected cells [49]. RIG-I specifically recognizes dsRNA with 5′-triphosphate motifs, a key feature of viral replication intermediates. This early detection by RIG-I is crucial for initiating a rapid antiviral response.

On activation, RLRs trigger downstream signaling by engaging MAVS. This MAVS-dependent signaling pathway leads to the robust production of type I and III IFNs, as well as inflammatory cytokines, through the activation of transcription factors like IRF3/7 and NF-κB. Studies demonstrate that RLR-mediated signaling is finely regulated through interactions with endogenous RNAs and host proteins, particularly those involved in stress responses and post-translational modifications. This regulation underscores the complexity and efficiency of the antiviral immune response mediated by RLRs [49].

While MDA5, LGP2, and NOD1 are crucial, the specific role of RIG-I in influencing IFN induction in response to SARS-CoV-2 remains uncertain or limited in some contexts, with questions persisting as to whether other sensors like RIG-I lack viral targets or are potentially inhibited by viral proteins [50]. Critically, in the lung, RLRs are the dominant sensors in alveolar and bronchial epithelial cells, the initial site of infection. This highlights that RLR signaling, particularly by MDA5, is essential for establishing the early antiviral state in the pulmonary environment. However, the virus is highly adept at counteracting this. Viral non-structural protein 5 (Nsp5) acts as a protease that cleaves RIG-I and induces MAVS degradation, effectively dismantling the core machinery for viral RNA detection and subsequent IFN signaling. This coordinated evasion mechanism, coupled with the action of ORF9b disrupting MAVS function, critically impairs the initiation of the type I IFN response, contributing to unchecked viral spread in the respiratory tract.

Structurally, RIG-I and MDA5 are similar and activate signaling through MAVS; however, RIG-I is more adept at detecting RNA with a 5′ triphosphate group, whereas MDA5 is more inclined to associate with long dsRNA [50]. SARS-CoV-2 infection induces IFNs, which bind to their receptors and activate JAK-STAT pathway. This leads to phosphorylation of JAK1, JAK2, JAK3, and TYK2, followed by activation of STAT1/STAT2 [51]. SARS-CoV-2 employs multiple mechanisms to interfere with signaling including the disruption of stress granule assembly, inhibition of RIG-I ubiquitination, and blockade of STAT1 and STAT2 phosphorylation [52]. Each of these pathways demonstrates a synergistic interplay among various viral proteins. Specifically, protein N, along with S, Orf7a, Orf7b, and Nsp6, facilitates the inhibition of STAT1 and STAT2 phosphorylation. Concurrently, the M protein, Orf3b, Nsp12, and Nsp15 impede the nuclear translocation of IRF3, thereby impairing the synthesis and signaling of IFN [53] (Table 2).

#### 2.2.2. MDA5 (Melanoma differentiation-associated gene 5)

MDA5 is a viral RNA sensor induced by SARS-CoV-2 [54]. Upon recognition of viral dsRNA, MDA5 is activated with K63-linked polyubiquitination and then triggers the recruitment of MAVS and activation of TBK1 and IKKα/β, subsequently leading to IRF3 and NF-κB phosphorylation [55]. Cytosolic RNA sensor MDA5 is required for type I and III IFN induction upon SARS-CoV-2 infection. Type I and III IFN induction further required MAVS and IRF3. In contrast, induction of IL6 and TNF was independent of the MDA5-MAVS-IRF3 axis [56]. Unlike RIG-I, MDA5 detects long stretches of dsRNA, typically formed as replication intermediates of RNA viruses, including SARS-CoV-2. Upon binding, MDA5 oligomerizes and recruits MAVS, activating downstream signaling that activate IRF3/7 and NF-κB for IFN production [57]. Knockout of MDA5 (but not RIG-I) significantly impairs IFN responses upon SARS-CoV-2 infection in certain cell lines [58]. Delayed or weak MDA5 activation might allow SARS-CoV-2 to evade early innate immunity [59].

#### 2.2.3. LGP2 (Laboratory of genetics and physiology 2)

LGP2, a helicase related to RIG-I and MDA5 but lacking the N-terminal CARD domain, can amplify the MDA5-mediated IFN response by enhancing MDA5 ability to form stable filaments on dsRNA. LGP2 also acts as an inhibitor of RIG-I signaling while activating MDA5 signaling [60]. RLRs, which include RIG-I, MDA5, and LGP2, are a family of innate immune receptors. They detect foreign RNA molecules produced during viral replication, stimulating inflammatory and antiviral responses by triggering signaling cascades involving the IRF and NF-κB pathways upon viral infection [49]. Unlike RIG-I and MDA5, LGP2 can’t directly bind to the downstream adaptor MAVS *via* CARD homotypic interactions because LGP2 completely lacks the N-terminal CARD domains.

### 2.3. Cytosolic DNA sensors

The cGAS-STING pathway, primarily involved in sensing self or pathogen dsDNA, also plays a role in host immunity against some ssRNA viruses [61]. Mechanistically, cGAS first catalyzes the production of cyclic GMP-AMP (cGAMP) from GTP and ATP. cGAMP then acts as a second messenger and binds to STING, resulting in its activation. STING proceeds to activate TBK1, which phosphorylates STING. Upon phosphorylation, STING recruits IRF3, which is then phosphorylated by TBK1. The phosphorylated IRF3 then dissociates from STING, dimerizes, and translocate to the nucleus to induce IFNs. Additionally, STING independently activates NF-κB leading to pro-inflammatory cytokine expression [62].

The cGAS-STING pathway becomes active in the later stages of SARS-CoV-2 infection. This occurs when infected cells display their spike protein on the surface, allowing them to bind to ACE2 receptors nearby, often uninfected epithelial cells [63]. This interaction triggers membrane fusion, leading to the formation of multinucleated syncytia. The fusion process causes cellular damage and nuclear instability, resulting in chromatin leakage into the cytoplasm. This cytoplasmic DNA is then detected by cGAS, which produces cGAMP to activate STING. Activation of the STING pathway leads to the production of IFNs and pro-inflammatory cytokines [62,64]. Persistent activation of this pathway contributes to a dysregulated immune response, excessive inflammation, tissue damage, and poor clinical outcomes. When combined with the detection of viral RNA and the activation of other PRRs, this pathway can synergistically amplify cytokine production [65]. cGAS-STING activity detected in lung samples from patients with COVID-19 with prominent tissue destruction is associated with type I IFN responses. A lung-on-chip model revealed that, infection with SARS-CoV-2 activates cGAS-STING signaling in endothelial cells through mitochondrial DNA release, which leads to cell death and type I IFN production [66].

### 2.4. NOD-like receptors (NLRs)

Nucleotide-binding oligomerization domain (NOD)-like receptors, also known as nucleotide-binding leucine-rich repeat receptors (NLRs) are cytosolic PRRs that detect PAMPs and DAMPs within the host cells, coordinating innate immune responses [10]. On activation, certain NLRs assemble inflammasomes, leading to caspase-1 activation, cytokine maturation (e.g., IL-1β, IL-18), and pyroptosis [67].

Caspase-1, in turn, cleaves and releases the pro-inflammatory cytokines IL-1β and IL-18. These cytokines significantly amplify the immune response, leading to severe inflammation and cytokine storms characteristic of severe COVID-19. Developing the lung-specific context, the alveolar macrophage (AM) is recognized as a key immune cell driving this process, as it is a major expressor of NLRP3 in the lower respiratory tract. In severe COVID-19, NLRP3 activation in AMs shifts from a protective function to a pathological one, serving as a central amplifier of the cytokine storm (e.g., IL-1β, IL-18). Additionally, DAMPs-such as HMGB1, ATP, and oxidized phospholipids-are released from dying epithelial and endothelial cells during severe tissue injury, which then further fuel NLRP3 activation and inflammation. Taken together, these findings establish the NLRP3 inflammasome as a key driver of SARS-CoV-2-induced immunopathology, making it a promising therapeutic target for mitigating cytokine-driven tissue damage, particularly in the lungs [68,69].

### 2.5. NLRP3 inflammasome

The NLRP3 inflammasome, a crucial cytosolic sensor, is activated by SARS-CoV-2 components like the S, E, orf3a, and N proteins [67,70]. The virus triggers this activation through three main mechanisms: potassium efflux, mitochondrial reactive oxygen species (ROS) production, and lysosomal damage [67,71]. This process initiates the assembly of the NLRP3 complex, which recruits and activates caspase-1. Caspase-1, in turn, cleaves and releases the pro-inflammatory cytokines IL-1β and IL-18 (Fig. 1). These cytokines significantly amplify the immune response, leading to the severe inflammation and cytokine storms characteristic of severe COVID-19 [72–76]. Additionally, DAMPs-such as HMGB1, ATP, and oxidized phospholipids-are released from damaged host cells and further fuel NLRP3 activation and inflammation [35,36]. Taken together, these findings establish the NLRP3 inflammasome as a key driver of SARS-CoV-2-induced immunopathology, making it a promising therapeutic target for mitigating cytokine-driven tissue damage, particularly in the lungs.

### 2.6. C-type lectin receptors (CLRs)

CLRs are carbohydrate-binding receptors highly expressed on myeloid cells, including DCs and macrophages, involved in pathogen recognition, antigen uptake, and immune regulation [77,78]. Several CLRs, like DC-SIGN (CD209) and MGL (CLEC10A), bind to glycosylated SARS-CoV-2-S protein, facilitating viral attachment and modulating immune responses [79–81]. DC-SIGN interacts with high-mannose glycans on the S protein, potentially enhancing viral uptake [82]. Similarly, MGL recognizes N-acetylgalactosamine (GalNAc)-containing glycans, influencing antigen presentation and T cell priming, thereby affecting the balance between tolerogenic and inflammatory responses [83]. CLRs engagement by SARS-CoV-2 can trigger signaling that modulates immunity and may impair antiviral responses [84,85].

## 3. Consequences of innate immune recognition in the lung

### 3.1. Type I interferon (IFN) response

Upon SARS-CoV-2 infection, host PRRs in the lung detect viral RNA, initiating intricate innate immune signaling cascades that culminate in the production of IFNs and pro-inflammatory cytokines. Among these, type I IFNs (primarily IFN-α, IFN-β, and IFN-ω) play a pivotal role, exhibiting the strongest antiviral effects against SARS-CoV-2 replication *in vitro*, despite Type II (IFN-γ) and Type III (IFN-λ) IFNs also demonstrating inhibitory capacities [86].

The induction of Type I IFNs, largely orchestrated by PRRs such as endosomal TLR3, TLR7/8 and cytosolic RLRs like MDA5, leads to the activation of IFN regulatory factors (IRF3/7) and NF-κB (Fig. 1). This drives the robust transcription and secretion of IFN-α/β, which then binds to the ubiquitously expressed IFN-α/β receptor (IFNAR), and initiate the JAK-STAT signaling pathway [87]. This culminates in the expression of hundreds of IFN-stimulated genes (ISGs), which collectively exert antiviral roles in limiting SARS-CoV-2 replication and spread within host cells. For instance, several ISGs, including lymphocyte antigen 6 complex, locus E (LY6E) [88,89] and cholesterol 25-hydroxylase (CH25) [90,91], inhibit SARS-CoV-2 entry by blocking spike protein-mediated membrane fusion with host cells. Nuclear receptor coactivator 7 (NCOA7) also restricts viral entry by targeting the endo-lysosomal pathway, relying on lysosomal protease activity, vesicle acidification, and degradation of endocytosed material to impair fusion [90,91]. Similarly, invariant chain CD74 impedes cathepsin-dependent viruses by restricting endosomal entry mechanisms [92]. IFN-induced transmembrane proteins (IFITMs) prevent viral fusion by embedding in endosomal membranes and disrupting the interaction between vesicle membrane protein-associated protein A (VAPA) and oxysterol-binding protein (OSBP). This leads to cholesterol accumulation that stiffens the endosomal membrane, thereby blocking the virus from fusing and releasing into the cytosol [93]. Mucins contribute by forming a dense glycosylated barrier that physically prevents viral attachment [94]. Death domain-associated protein 6 (DAXX) exerts antiviral effects early in viral entry by relocating to the cytoplasm to inhibit replication [95]. Zinc finger antiviral protein (ZAP) binds CpG-rich regions in viral RNA, directing them to degradation pathways involving tripartite motif-containing 25 protein (TRIM25) and KH and NYN domain-containing protein (KHNYN) [96]. Beyond direct viral inhibition, a robust IFN response is also crucial for bridging innate and promoting adaptive immunity by facilitating antigen presentation and T cell activation.

Patients with severe COVID-19 frequently exhibit defects or delays in their type I IFN responses and subsequent downregulation of ISGs [47]. This impairment is largely attributed to the sophisticated viral evasion mechanisms employed by SARS-CoV-2 multifunctional proteins to antagonize the host IFN response [97]. These evasion strategies have been broadly categorized into five classes: minimizing and masking inflammatory RNA, blocking host recognition, blocking IFN signaling, blocking nuclear transport, and shutting off host translation [98].

Interestingly, despite SARS-CoV-2’s various strategies to block PRR pathways, production of cytokines *via* NF-κB independent mechanisms remains active, while IFN-I or IFN-III, which require NF-κB and IRF3 and/or IRF7 signaling, remain impaired [99]. Additionally, inhibition of NF-κB has been shown to decrease cytokine production but paradoxically increase SARS-CoV-2 replication, indicating that the transcriptional output of NF-κB can be beneficial to the virus [100].

### 3.2. Viral modulation of the innate response: mechanisms of antagonism

SARS-CoV-2 deploys an arsenal of proteins that directly interferes with host innate immune signaling and specifically targets the Type I IFN pathway. This multifaceted evasion strategy is central to establish infection, dampen antiviral defenses, and contribute to severe disease outcomes. Understanding these mechanisms is paramount for deciphering COVID-19 pathogenesis and identifying therapeutic vulnerabilities.

Viral structural proteins are among the first to encounter the host immune system and have evolved distinct strategies to subvert initial defenses. The S protein, beyond its role in host cell entry, actively engages with IRF3, and directly interferes with the induction of antiviral responses [101]. The M protein stands out for its critical role in evading innate immunity, exhibiting multifaceted antagonism. It potently inhibits the TRAF complex, which is essential for NF-κB promoter activation and subsequent IFN transcription [102–104]. Furthermore, the M protein’s interaction with MAVS disrupts downstream IFN signaling, and its inhibition of STAT1 phosphorylation underscores a broad interference with ISG induction [105]. The N protein directly targets key components of the early antiviral response by obstructing the initiation of the RIG-I signaling pathway through TRIM25 inhibition [106]. More broadly, it prevents the phosphorylation and nuclear translocation of STAT1, STAT2, and IRF3, effectively impeding both IFN and ISG responses [51]. Curiously, while inhibiting MAVS aggregation, the N protein also promotes inflammasome activation, suggesting a complex interplay that may contribute to dysregulated inflammation [107].

The array of non-structural proteins (Nsps) and accessory open reading frames (ORFs) encoded by SARS-CoV-2 collectively represents a formidable force against host immunity, often exhibiting overlapping or synergistic functions that underscore the virus’s extensive adaptive capacity. To achieve mechanistic clarity, we focus on a few key antagonists that form the core "Axis of Immune Antagonism": Nsp1 is a potent antagonist, blocking the phosphorylation of IRF3, IRF7, STAT1, and cJun, and directly inhibiting both IFN and NF-κB promoters [108,109]. Its primary mechanism is to suppress host protein translation and degrade host transcripts lacking a 5′ viral leader sequence, further highlighting a global dampening of antiviral gene expression. Nsp3 also impedes cytokine production by inhibiting IRF3 phosphorylation, thereby preventing its nuclear translocation and antagonizing type I IFN activity [110]. The Nsp5 protease (3CLpro) demonstrates a critical point of viral intervention by proteolytically cleaving components of the RLR pathway, specifically RIG-I, and inducing MAVS degradation, dismantling the core machinery for viral RNA detection and subsequent IFN signaling [111](Fig. 1). Other Nsps contribute to this broad suppression: nsp8 and nsp9 disrupt protein trafficking by binding to the signal recognition particle (SRP), further suppressing the type I IFN response [109]. Furthermore, nsp6 and nsp13 collectively interact with intermediaries in the MAVS-IRF3 signaling cascade, thereby limiting IRF3 activation [112,113]. They also inhibit the phosphorylation of STAT1 and STAT2, with nsp13 also obstructing the nuclear translocation of NF-κB [113]. Notably, a concerted effort by nsp13, nsp14, nsp15, and ORF6 impedes the nuclear translocation of IRF3, highlighting a multi-pronged attack on this transcription factor [114]. Beyond direct signaling interference, nsp14, nsp15, and nsp16 are involved in modifying viral RNA to evade recognition by RIG-I and MDA-5, and facilitate the lysosomal degradation of the IFNAR receptor, showcasing mechanisms of immune evasion [108]. Nsp16 also interacts with the spliceosome, disrupting mRNA splicing and dampening the IFN response [110].

Finally, accessory proteins (ORFs) contribute to immune evasion through diverse and often multifunctional roles beyond direct IFN antagonism. While possessing multiple roles, Orf3a is a major accessory protein that promotes pyroptosis and IL-1α\β release by driving the activation of the NLRP3 inflammasome. Orf3a not only inhibits STAT1 phosphorylation but also activates the NLRP3 inflammasome, prevents phagolysosome fusion, and triggers the extrinsic apoptosis pathway [110,113,115]. Orf3b acts as a direct antagonist to type I IFN activity [116]. Orf6 broadly obstructs the nuclear translocation of both IRF3 and the STAT1 complex, inhibiting the MHC class I pathway. Its specific mechanistic depth lies in physically binding to the nucleoporin Nup98 component of the nuclear pore complex, thereby preventing the expression of IFN-stimulated genes (ISGs). It also binds to the IFN-inducible nuclear export complex (Nup98 and Rae1), preventing mRNA nuclear export, and, in concert with Orf3b and Orf8, inhibits ISRE-mediated type I IFN production [81,112]. Orf7a hinders STAT2 nuclear translocation, reduces phagolysosomes acidity, and interacts with monocytes, diminishing their antigen-presenting capabilities [110, 113,117]. Orf7b suppresses the phosphorylation of both STAT1 and STAT2 [113]. Orf8 directly engages with MHC class I proteins on the endoplasmic reticulum membrane, facilitating their degradation through autophagic pathways [118]. It also acts as a secreted mimic of IL-17A, interfacing with IL-17 receptors on monocytes to upregulate genes involved in fibrosis signaling, coagulation dysregulation, and inflammatory responses [119]. Orf9b interacts with Tom70, disrupting MAVS function and type I IFN expression. Conversely, Orf9c upregulates IL-6 signaling while simultaneously impairing IFN signaling [81,120]. Lastly, Orf10 promotes mitophagy, leading to the degradation of MAVS [121]. Table 2 summarizes key SARS-CoV-2 viral proteins and their targeted host immune components or pathways, along with their respective effects on host immunity. The sheer number and diverse mechanisms of these viral protein-mediated interferences underscore the challenge SARS-CoV-2 poses to an effective host innate immune response, directly contributing to delayed or dampened IFN production and, consequently, severe COVID-19 pathology.

The concerted effort of these and other Nsps (e.g., Nsp3 inhibiting IRF3 phosphorylation; Nsp13 obstructing NF-κB nuclear translocation; Nsp14/15/16 modifying viral RNA to evade RLRs) and ORFs (e.g., Orf3b, Orf8, Orf9b/c) underscores a multi-pronged attack on the host’s antiviral defense. The sheer number and diverse mechanisms of these viral protein-mediated interferences underscore the challenge SARS-CoV-2 poses to an effective host innate immune response, directly contributing to delayed or dampened IFN production and, consequently, severe COVID-19 pathology.

### 3.3. Integration of circulating variants with innate immune evasion

The evolutionary trajectory of SARS-CoV-2 is intrinsically linked to the optimization of innate immune evasion strategies. The emergence of Variants of Concern (VOCs) demonstrates that key mutations can enhance the virus’s capacity to suppress host defenses, directly influencing transmissibility and pathogenesis.

Delta Variant (B.1.617.2): This VOC often displayed significantly lower Type I IFN production in infected cells compared to ancestral strains in *in vitro* models. This enhanced immune suppression was mechanistically linked to increased antagonism of the IFN promoter by the Nucleocapsid (N) protein, potentially through optimized binding or expression, a trait that contributed to the variant’s greater viral loads and heightened severity during its wave of circulation [122].

Omicron (BA.X) Lineages: These highly transmissible lineages exhibit superior innate immune evasion in the upper respiratory tract compared to earlier strains. This increased capacity for evasion, particularly against the RLR-MAVS pathway, is hypothesized to be linked to subtle but critical mutations that alter the expression, stability, or efficiency of key innate immune antagonists (e.g., Nsp5/3CLpro, ORF6, ORF9b). This faster suppression of the initial IFN response in the upper airways allows for rapid viral replication and shedding, which directly contributes to the observed high transmissibility and high rates of reinfection [123].

Understanding these variant-specific evasion mechanisms is crucial for developing pan-viral antiviral therapies that target conserved weak points in the viral antagonism machinery rather than just the highly mutable Spike protein.

### 3.4. Pro-inflammatory cytokine and chemokine production

A rapid and highly dynamic cascade of pro-inflammatory cytokines and chemokines is triggered in response to PRR recognition of viral components by epithelial and innate immune cells. This local response is initially crucial for controlling viral replication, recruiting essential immune cells, and initiating tissue repair [124]. However, a critical aspect of SARS-CoV-2 pathogenesis is the dysregulation of this response, which allows excessive systemic inflammation, widespread tissue damage, and, in severe cases, the life-threatening phenomenon known as a "cytokine storm" [73]. Key mediators contributing to this complex inflammatory landscape include TNF-α, IL-1β, IL-6, IL-10, IL-8, and MCP-1, with each playing distinct yet interconnected roles in shaping the lung immune environment and influencing disease progression [125]. Fig. 2 summarizes the immune responses triggered by SARS-CoV-2.

Rapid and sustained elevation of TNF-α is a hallmark of severe COVID-19. TNF-α binds primarily to TNF receptor 1 (TNFR1) on various cell types, triggering distinct downstream signaling pathways *via* TRADD and TRAF2, ultimately activating NF-κB and MAPK pathways that promote the transcription of other pro-inflammatory cytokines, including IL-6 [126–128]. Prolonged TNF-α action, especially through TNFR2-mediated canonical and noncanonical NF-κB activation, contributes to amplified inflammation, potentially exacerbating cytokine storm severity [129].

In SARS-CoV-2 infection, TNF-α-mediated inflammation drives bronchial hyperresponsiveness due to elevated cytokine levels and neutrophil infiltration in the airway epithelium [130]. These neutrophils release matrix metalloproteinase-9 (MMP-9), a protease linked to extensive tissue remodeling and lung fibrosis [131]. Furthermore, elevated TNF-α levels are consistently observed in COVID-19 patients with comorbidities such as obesity, hypertension, and cardiovascular disease (CVD), where it fuels heightened inflammation, endothelial dysfunction, increased thrombosis risk, and atherosclerotic plaque destabilization, elevating the risk of acute cardiovascular events [132]. Its persistent elevation even after viral clearance is also strongly implicated in post-acute sequelae of COVID-19 (long COVID), potentially underlying chronic symptoms such as dyspnea, fatigue, and cognitive disturbances [133–135]. In long COVID, various types of mitochondrial dysfunction have been observed such as fatty acid metabolism, loss of mitochondrial membrane potential, abnormal level of mitochondrial proteins along with aberrant accumulation of viral S and N proteins in the central nervous system [136,137].

IL-6 is a pleiotropic cytokine widely recognized as a central driver and reliable biomarker of severe COVID-19, with elevated levels strongly correlating with disease severity and poor prognosis [138]. Produced by diverse cell types, including monocytes, macrophages, and endothelial cells, IL-6 contributes to the cytokine storm [139]. Mechanistically, SARS-CoV-2 itself appears to directly enhance IL-6 production, as its N protein has been shown to augment NF-κB activation and promote IL-6 expression in human AECs [140], highlighting a direct viral contribution to this key inflammatory mediator.

Interleukin-8 (IL-8), or CXCL8, is primarily responsible for the robust recruitment of neutrophils to sites of infection and inflammation in the lung. Produced by various cells in response to PRR activation *via* NF-κB and AP-1 pathways, IL-8 binds to CXCR1 and CXCR2 on neutrophils, triggering their chemotaxis, degranulation, ROS production, and NETosis [141]. In SARS-CoV-2 infection, IL-8 levels are elevated, particularly in severe cases [142]. This increase is partly driven by viral activation of NF-κB, notably through SARS-CoV-2 nonstructural protein 14 (Nsp14), which enhances IL-8 transcription [143]. The massive influx of neutrophils orchestrated by IL-8 leads to the release of destructive mediators such as ROS, proteases, and neutrophil extracellular traps (NETs), all contributing to severe alveolar damage, vascular leakage, and ultimately, pulmonary fibrosis. Thus, IL-8 is a critical mediator of acute lung injury (ALI) and acute respiratory distress syndrome (ARDS) in COVID-19 [144,145].

Monocyte Chemoattractant Protein-1 (MCP-1), also known as CCL2, plays a crucial role in orchestrating the inflammatory cellular infiltration by recruiting monocytes to sites of infection or injury. Produced by monocytes, endothelial cells, macrophages, and fibroblasts in response to PRR activation or pro-inflammatory cytokines, MCP-1 binds CCR2 on classical monocytes (CD14 ++CD16-), guiding their chemotaxis to inflamed tissues [146]. Once recruited, these monocytes differentiate into macrophages or DCs, which then release additional cytokines like TNF-α and IL-6, thereby amplifying the inflammatory cascade and contributing to increased vascular permeability. Given its central role in monocyte recruitment and subsequent inflammation, MCP-1 levels have been established as significant biomarkers of disease severity in COVID-19 [147].

The unchecked production and synergy of these and other inflammatory mediators culminate in cytokine storm [29]. In COVID-19, this phenomenon is directly associated with diffuse alveolar damage, vascular leakage, thrombo-inflammation, and multi-organ dysfunction [148]. Elevated levels of TNF-α, IL-6, IL-8, and MCP-1 drive continuous recruitment and activation of neutrophils, monocytes, and lymphocytes, leading to widespread endothelial activation, disruption of epithelial barriers, and extensive tissue destruction, particularly in the lungs [149]. The amplification loops between cytokine production and immune cell recruitment, coupled with delayed viral clearance or active immune evasion by SARS-CoV-2, create a self-perpetuating cycle of inflammation. The severity of this cytokine storm directly correlates with poor clinical outcomes and is a major driver of ARDS, pulmonary fibrosis, and multi-organ failure in critically ill COVID-19 patients [150]. This highlights the urgent need for therapeutic strategies that can precisely modulate, rather than indiscriminately suppress, these inflammatory responses.

### 3.5. Activation of immune cells

SARS-CoV-2 infection typically initiates in lung epithelial cells, where viral entry is mediated by ACE2 binding and S protein cleavage by TMPRSS2. This initial viral presence, along with viral RNA detection by PRRs on both epithelial and resident immune cells (including AMs and DCs), triggers critical downstream signaling pathways like NF-κB and IRF3/7 [151]. This early recognition is pivotal, orchestrating the rapid production of pro-inflammatory cytokines (e.g., IL-6, IL-1β, TNF-α) that induce fever and are essential for recruiting additional immune cells to the infection site. While Type I and III IFNs (IFN-α/β and IFN-λ) are produced, a notable and consistent finding in COVID-19 pathogenesis is the early suppression or delay of this crucial IFN response by SARS-CoV-2, occurring *via* mechanisms discussed above and often correlating with disease severity [152].

Resident immune cells, particularly AMs, serve as first responders. Beyond their role in cytokine and chemokine production (such as CXCL8/IL-8 and CCL2, macrophages actively engage in phagocytosis, engulfing viral particles and cellular debris from dying cells [153]. Simultaneously, DCs, APCs, capture viral antigens and undergo maturation. This maturation, characterized by upregulation of MHC and co-stimulatory molecules, enables their migration to lymph nodes to initiate adaptive immunity. A specialized subset, plasmacytoid dendritic cells (pDCs), are recognized as the primary producer of large amounts of Type I IFNs (IFN-α and IFN-β); however, similar to the broader IFN response, their function in severe SARS-CoV-2 infection is often impaired or delayed due to viral immune evasion, representing a significant gap in the early antiviral response [75].

The initial chemokine surge, particularly IL-8 and CCL2, orchestrates the swift influx of neutrophils and monocytes. Monocytes differentiate into tissue-resident macrophages or DCs, amplifying the inflammatory response and further secreting CCL2 to recruit more immune cells [151]. Neutrophils, by releasing destructive NETs (webs of DNA coated with antimicrobial enzymes designed to trap and neutralize the virus) and producing ROS, which directly damage viral components and infected host cells [154,155]. While these mechanisms are protective, excessive or uncontrolled activation of these immune cells, macrophages, DCs, monocytes, and particularly neutrophils, lead to detrimental consequences. This overactivation precipitates uncontrolled cytokine production and severe tissue injury, directly contributing to the cytokine storm and the development of ARDS, a hallmark of severe COVID-19 (ARDS) [148,156]. The precise triggers and individual cell contributions to this pathogenic overactivation remain a key area for further elucidation.

### 3.6. Crosstalk with adaptive immunity

The innate immune recognition of SARS-CoV-2 and the resulting cytokine and chemokine milieu fundamentally prime and shape the subsequent adaptive immune response, determining its efficacy and potential for pathology. This intricate crosstalk is mediated by several key immune cell populations. Conventional DCs are unequivocally considered the most effective APCs for initiating robust adaptive immunity [157]. On viral encounters, DCs process viral proteins into antigenic peptides, which are then presented on their surface MHC. MHC class I presentation activates CD8 + cytotoxic T cells, crucial for killing infected cells, while MHC class II presentation activates CD4 + helper T cells that coordinate broader immune responses [158]. This process involves DC maturation, upregulation of MHC and co-stimulatory molecules, and subsequent migration to secondary lymphoid organs to activate naive T cells. The efficiency of this priming step is critical, and any viral interference at this stage could significantly delay effective adaptive immunity.

pDCs are widely acknowledged as the primary producers of type I IFNs (IFN-α/β) during infection [159]. Abundant Type I IFN production is not only directly antiviral but also crucial for promoting B cell activation and differentiation into antibody-producing plasma cells, thus supporting humoral immunity. However, a consistent observation in severe SARS-CoV-2 infection is the reduction and functional impairment of pDCs, leading to markedly decreased Type I IFN production compared to moderate disease [160,161]. This defect in early IFN signaling from a critical source likely contributes to delayed viral clearance and compromised adaptive immune priming.

NK cells play a dual role, bridging innate and adaptive immunity. Early in SARS-CoV-2 infection, NK cells rapidly identify and kill infected cells independent of antigen presentation *via* MHC class I, enabling swift containment of viral spread [162]. Furthermore, NK cells are a major source of IFN-γ, which is critical for enhancing the adaptive immune response by increasing MHC class I and II expression on APCs, thereby improving their ability to activate CD8 + cytotoxic and CD4 + helper T cells [163]. A notable area of ongoing investigation and conflicting results pertains to NK cell dynamics in the lungs of COVID-19 patients, with some studies report a decrease in resting NK cells [164], while others observe increased NK cell numbers in severe disease [160]. These disparities highlight the complex and dynamic interplay of NK cells during SARS-CoV-2 infection and underscore a gap in a comprehensive, unified understanding of their precise contribution to both protective immunity and immunopathology, necessitating further targeted research. To further illustrate the viral strategies for immune evasion (Table 2).

## 4. Lessons learned from COVID-19: implications for respiratory health and therapeutics

The COVID-19 pandemic and the worldwide focus of research on this disease have profoundly advanced our understanding of innate immune responses to respiratory viral infections, particularly within the lung. Insights from SARS-CoV-2 pathogenesis underscore the critical balance for effective antiviral immunity and highlight the detrimental consequences of dysregulated innate responses. These lessons are now directly informing the development of novel therapeutic strategies aimed at modulating innate immunity.

### 4.1. Aberrant innate immune responses and disease severity

Concurrently, severe COVID-19 is defined by a profound hyperinflammatory state, or “cytokine storm”. This hyperinflammation, driven by direct viral PRR activation and host DAMPs, creates a self-amplifying loop that perpetuates lung tissue damage, vascular leakage, and thrombo-inflammation, highlighting the strong correlation between elevated cytokine levels and poor clinical outcomes, including ARDS, multi-organ failure, and mortality [125,139,142,147,150]. Beyond acute severity, innate immune dysregulation also underpins long-term respiratory complications and post-acute sequelae of COVID-19 (Long COVID). Persistent elevation of inflammatory mediators, such as TNF-α, even after viral clearance, suggests ongoing immune activation contributing to chronic symptoms like dyspnea and fatigue [133–135]. The precise mechanisms linking acute innate immune dysregulation to chronic sequelae remain an active area of investigation and a significant knowledge gap, requiring further research for targeted therapies.

### 4.2. Targeting innate immunity for therapeutics

Insights into aberrant innate immune responses have spurred interest in modulating these pathways for therapeutic benefit, aiming to either boost protective immunity or dampen detrimental hyperinflammation.

#### TLR agonists/antagonists:

Given the dual role of TLRs in antiviral defense and hyperinflammation, TLR modulation is being actively explored as a potential therapeutic strategy to enhance protective immunity. TLR agonists (e.g., TLR3 agonists like Poly ICLC, TLR7/8 agonists like R848) could enhance early antiviral responses by promoting IFN production, particularly in patients with delayed IFN responses [165]. However, the risk of exacerbating inflammation, especially in later stages, necessitates careful timing and patient stratification. Conversely, TLR antagonists could dampen hyperinflammation driven by excessive TLR2 and TLR4 activation [18,26] (Fig. 2). The challenge lies in selectively inhibiting pathogenic inflammation without compromising essential antiviral signaling, indicating a clear need for more specific TLR modulators.

#### RIG-I/MDA5 modulators:

Strategies to boost the activation of these cytosolic RNA sensors that function in early detection and IFN induction could enhance early viral detection and promote a more robust initial IFN response, potentially preventing viral escape. Challenges include the complex interplay among PRRs, and the sophisticated evasion mechanisms employed by SARS-CoV-2 (e.g., Nsp5, ORF9b) [111,120]. A critical research gap lies in the development of highly effective and specific RIG-I/MDA5 modulators that can not only enhance antiviral responses but also overcome viral mechanisms of immune evasion.

#### NLRP3 inflammasome inhibitors:

Inhibiting NLRP3 activation is a promising therapeutic avenue for mitigating the cytokine storm and associated tissue damage. While several inhibitors such as mefenamic acid, indomethacin and metformin are under investigation, optimizing dosage, timing, and identifying appropriate patient populations are critical challenges requiring further research (Fig. 2).

#### Antiviral agents and interferon-based therapies:

Clinical outcomes for IFN treatment in COVID-19 have been mixed and largely inconclusive for Type I IFNs. Early in the pandemic, repurposed drugs such as the protease inhibitors lopinavir/ritonavir and Type I interferon were explored due to promising in vitro activity against SARS-CoV-2 [166]. Initial, small-scale trials suggested potential benefits; however, subsequent large, randomized controlled trials (e.g., WHO Solidarity Trial) concluded that the combination of lopinavir/ritonavir with interferon, or their use alone, demonstrated no significant clinical benefit in reducing mortality for hospitalized COVID-19 patients, resultingly their use has largely been discontinued in current standard clinical practice. This outcome underscores the challenge of translating *in vitro* efficacy to *in vivo* clinical benefit, particularly when viral evasion mechanisms, such as ORF6 antagonizing IFN signaling, are highly active [167]. Consequently, the focus has shifted to direct-acting antivirals (e.g., Remdesivir, Paxlovid) that specifically target key viral replication enzymes (e.g., RdRp, Nsp5) to effectively reduce viral load [168] (Fig. 2).

#### Immunomodulatory agents

##### Established clinical cytokine blockers:

Tocilizumab, an IL-6 receptor blocker, is a clinically established therapy that demonstrably improves outcomes in hospitalized patients with rapidly progressing respiratory failure and elevated inflammatory markers such as C-reactive protein (CRP) [169]. Similarly, Janus Kinase (JAK) inhibitors (e.g., Baricitinib) are clinically established agents for their anti-inflammatory effects by blocking cytokine signaling downstream of the receptor and are recommended for use in combination with corticosteroids in specific hospitalized patient subsets. IL-6, a key driver of excessive inflammation, is a major therapeutic target, tocilizumab, sarilumab and siltuximab are approved IL-6/IL-6R inhibitors [170] (Fig. 2).

##### Experimental and preclinical immunomodulation:

Other immunomodulatory approaches are currently under investigation. Targeting the NLRP3 inflammasome directly with inhibitors or exploring novel antibodies against TNF-α and specific chemokines (e.g., IL-8/CXCL8) are areas of active basic and translational research. These strategies hold preliminary promise for managing specific aspects of pulmonary pathology but currently lack the extensive clinical validation necessary for broad inclusion in standard treatment guidelines [169,171,172]. This experimental data must be strictly separated from established clinical efficacy and is often limited to in vitro or small animal model studies. Table 3 presents an overview of potential therapeutic targets within innate immune recognition pathways, highlighting various therapeutic categories, specific examples, their mechanisms of action, and important challenges or considerations for clinical application.

### 4.3. Future directions and research gaps

Despite significant progress, several areas require further investigation to fully harness innate immunity for respiratory health and therapeutic interventions. A significant research gap lies in elucidating the specific roles of different PRRs in various lung cell types (e.g., alveolar epithelial cells, macrophage subsets). Understanding cell-specific PRR expression and signaling nuances will enable more targeted interventions.

Further research is also needed to comprehensively map the mechanisms of viral immune evasion beyond enumeration, including identifying novel viral antagonists and how variants alter these strategies. Such knowledge is therefore crucial for developing pan-viral therapeutics.

Finally, the development of targeted immunotherapies remains a high priority. This involves designing compounds that precisely modulate specific innate immune pathways, either by enhancing protective responses or dampening pathogenic ones, all without causing broad immunosuppression. Future research should focus on several key areas. First, biomarker discovery is crucial to identify reliable indicators that can guide the timing and selection of immunomodulatory therapies. Second, exploring combination therapies that pair antivirals with innate immune modulators could lead to synergistic effects. Third, host-directed therapies that target host factors essential for viral replication or immune dysregulation need to be developed. Lastly, it is vital to continue investigations into innate immune dysregulation in Long COVID to identify potential targets for intervention.

Addressing these critical research gaps will be instrumental in translating our understanding of innate immunity into effective clinical strategies, helping us better manage both current and future respiratory viral pandemics.

## 5. Conclusion

The COVID-19 pandemic has profoundly underscored the indispensable role of the innate immune system as the first line of defense against respiratory viruses like SARS-CoV-2. This review highlights the crucial involvement of various PRRs in the lungs, including TLRs such as TLR2, TLR3, TLR4, TLR7/8; cytosolic RNA sensors like RIG-I and MDA5; and inflammasome-forming NLRs such as NLRP3, along with CLRs. Each of these PRRs, upon recognizing viral components or host-derived damage signals, initiates distinct yet interconnected downstream signaling cascades.

The consequences of this innate immune recognition are dual edged: while essential for triggering antiviral Type I IFN responses and recruiting immune cells for viral clearance, their dysregulation leads to severe pathology. Concurrently, exaggerated pro-inflammatory cytokine and chemokine production (e.g., TNF-α, IL-6, IL-8, MCP-1), often culminating in a "cytokine storm," drives extensive lung damage, ARDS, and multi-organ dysfunction. This aberrant response also contributes to the persistent inflammation and immune dysregulation observed in long COVID. Understanding these initial host-virus interactions is paramount for comprehending the complex pathogenesis of COVID-19 and, more broadly, for developing effective therapeutic interventions for lung and respiratory health against current and future viral threats. The intricate balance between robust antiviral immunity and controlled inflammation is key.

Looking forward, future research must focus on addressing critical gaps in knowledge. This includes elucidating the precise, cell-type-specific roles of different PRRs within the heterogeneous lung environment and comprehensively mapping the sophisticated mechanisms by which SARS-CoV-2 and other respiratory viruses evade or manipulate innate immune pathways. Such targeted understanding will be instrumental in designing precision immunotherapies that can selectively enhance protective responses (e.g., early IFN induction) or mitigate pathogenic inflammation (e.g., specific inflammasome inhibition), ultimately translating into more effective strategies for managing respiratory viral diseases and improving patient outcomes. Ultimately, deciphering how innate sentinels operate within the unique lung microenvironment, from epithelial surfaces to alveolar niches, will be pivotal not only for combating SARS-CoV-2 but also for safeguarding respiratory health against future viral threats.

## Figures and Tables

**Table 1:** Key Pattern Recognition Receptors (PRRs) in SARS-CoV-2 Recognition.

PRR	Cellular Compartment	Key Ligands	Downstream Signaling Pathway	Primary Cytokine/IFN Outcomes	References

TLR2	Surface	Viral proteins (E/M/N) + DAMPs	NF-κB	IL-6, TNF-α, IL-1β, IL-8	[17,18] [20,173]
TLR3	Endosome	dsRNA	IRF3/7	Type I/III IFNs	[21,23,174,175,176] [25]
TLR4	Surface	S protein + DAMPs	NF-κB	IL-6, TNF-α, IL-1β, IL-8	[26–28,177]
TLR7/8	Endosome	ssRNA	MyD88-dependent signaling → NF-κB & IRF3/7	Pro-inflammatory Cytokines & Type I/III IFNs	[38–42] [43,44,46,48]
CLRs (e.g., DC-SIGN)	Surface	S protein glycans	Modulate immune response/Facilitate viral entry		[79,84,85,178]
MDA5	Cytosol	Long dsRNA	MAVS→RF3/7	Type I/III IFNs	[54–59]
NLRP3	Cytosol	DAMPs/viral RNA indirectly (ORF3a, N, E)	Inflammasome complex → Caspase-1	IL-1β, IL-18	[67,71,72,75] [107,115]
RIG-I	Cytosol	5’-triphosphate RNA/dsRNA	MAVS→RF3/7	Type I/III IFNs	[49,16,50–53],179, 180, [167]

**Table 2:** SARS-CoV-2 Viral Proteins and Their Immune Evasion Targets.

SARS-CoV-2 Viral Protein	Targeted Host Component/Pathway	Effect on Host Immunity	References

E (Envelope)	NLRP3 inflammasome	Promotes Inflammasome activation	[67,70]
M (Membrane)	MAVS, STAT1, TRAF complex	Broad inhibition of NF-κB, IFN signaling, and ISG expression	[102–105]
N (Nucleocapsid)	IFN pathway, IRF3/7, MAVS, NLRP3 inflammasome, RIG- I/TRIM25, STAT1/2	Suppresses IFN/ISG induction; promotes Inflammasome activation	[51,106,107]
Nsp1	Host mRNA, IFN pathway, IRF3/7, MAVS, STATs	Blocks promoter activity; global suppression of host translation/gene expression	181, [108,109]
Nsp13	IRF3/NF-κB, STAT1/2	Limits IRF3 activation; obstructs NF-κB nuclear translocation	[113,114]
Nsp14/15/16	IFNAR, Spliceosome, Viral RNA	Modifies RNA (evades MDA5/RIG-I); degrades IFNAR; disrupts mRNA splicing	[108,110,114]
Nsp3	IRF3	Inhibits IRF3 phosphorylation; antagonizes Type I IFN	[110]
Nsp5	MAVS, RIG-I	Proteolytically cleaves RIG-I; degrades MAVS, dismantling IFN machinery	[111]
Nsp6	MAVS-IRF3, STAT1/2	Limits IRF3 activation; inhibits STAT phosphorylation	[112,113]
Nsp8/9	Protein Trafficking	Disrupts protein transport; suppresses Type I IFN production	[109]
ORF3a	NLRP3 inflammasome, STAT1	Inhibits STAT1; activates NLRP3 Inflammasome; promotes apoptosis	[115,110], [113]
ORF6	IFN pathway, IRF3/7, MAVS Nup98/Rae1, STAT1	Obstructs nuclear translocation of key transcription factors; blocks mRNA nuclear export	[112,81,108],182, [114]
ORF8	IL-17A Receptor, MHC I	Facilitates MHC I degradation; promotes inflammatory/fibrotic signaling	[118,119]
ORF9b/10	MAVS (via Tom70/Mitophagy)	Disrupts MAVS function; degrades MAVS, impairing Type I IFN expression	[81,120,121]
S (Spike)	IRF3	Interferes with early antiviral response induction	[101]

**Table 3:** Overview of Potential Therapeutic Targets within Innate Immune Recognition Pathways.

Therapeutic Target Category	Specific Targets	Purpose/Mechanism of Action	Challenges/Considerations	References

TLR Modulators	TLR3 agonists (e.g., Poly ICLC), TLR7/8 agonists (e.g., R848)	Enhance early antiviral responses promoting IFN production	Risk of exacerbating inflammation	[18,26]
	TLR2 and TLR4 antagonists	Dampen hyperinflammation driven by excessive TLR2 and TLR4 activation	Challenge in selectively inhibiting pathogenic inflammation without compromising essential antiviral signaling	[18,26]
RIG-I/MDA5 Modulators	RIG-I/MDA5 activators	Boost early viral detection and promote a more robust initial IFN response, potentially preventing viral escape	Complex interplay among PRRs and the virus’s sophisticated evasion mechanisms (e.g., Nsp5, ORF9b)	[111,120]
NLRP3 Inflammasome Inhibitors	NLRP3 inhibitors	Mitigate cytokine storm and associated tissue damage by inhibiting IL-1β and IL-18 release	Optimizing dosage, timing, and identifying appropriate patient populations are critical challenges	[70,72]
Interferon-based Therapies	Type I IFNs (IFN-α, IFN-β)	Provide antiviral activity against SARS-CoV-2	Mixed and largely inconclusive clinical outcomes for Type I IFNs; early administration is crucial; viral antagonism of IFN signaling (e.g., ORF9b targeting NEMO) is a barrier, suggesting combination therapies may be more effective	[86,166, 167]

This Table conveys the complexity of the innate immune response to SARS-CoV-2 and the strategic points where therapeutic interventions can be applied to rebalance the host-virus interaction.
